# Complex sequence organization of heterochromatin in the holocentric plant *Cuscuta europaea* elucidated by the computational analysis of nanopore reads

**DOI:** 10.1016/j.csbj.2021.04.011

**Published:** 2021-04-22

**Authors:** Tihana Vondrak, Ludmila Oliveira, Petr Novák, Andrea Koblížková, Pavel Neumann, Jiří Macas

**Affiliations:** a Biology Centre, Czech Academy of Sciences, Institute of Plant Molecular Biology, Branišovská 31, České Budějovice CZ-37005, Czech Republic; b University of South Bohemia, Faculty of Science, České Budějovice, Czech Republic

**Keywords:** Satellite DNA, Heterochromatin, Oxford Nanopore sequencing, Fluorescence *in situ* hybridization, Holocentric chromosomes, LINE elements

## Abstract

Repeat-rich regions of higher plant genomes are usually associated with constitutive heterochromatin, a specific type of chromatin that forms tightly packed nuclear chromocenters and chromosome bands. There is a large body of cytogenetic evidence that these chromosome regions are often composed of tandemly organized satellite DNA. However, comparatively little is known about the sequence arrangement within heterochromatic regions, which are difficult to assemble due to their repeated nature. Here, we explore long-range sequence organization of heterochromatin regions containing the major satellite repeat CUS-TR24 in the holocentric plant *Cuscuta europaea*. Using a combination of ultra-long read sequencing with assembly-free sequence analysis, we reveal the complex structure of these loci, which are composed of short arrays of CUS-TR24 interrupted frequently by emerging simple sequence repeats and targeted insertions of a specific lineage of LINE retrotransposons. These data suggest that the organization of satellite repeats constituting heterochromatic chromosome bands can be more complex than previously envisioned, and demonstrate that heterochromatin organization can be efficiently investigated without the need for genome assembly.

## Graphical abstract

**Figure:**
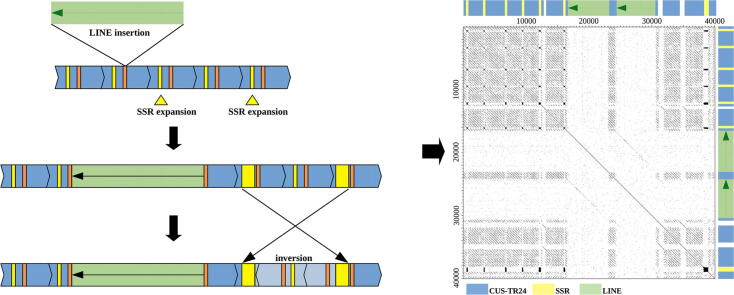

## 1 Introduction

Heterochromatin is a tightly packed, fundamental form of chromatin organization in eukaryotic nuclei exhibiting a unique combination of post-translational histone modifications [1], [49]. In higher plants, cytologically defined constitutive heterochromatin is mostly associated with large tracks of highly repetitive satellite DNA (satDNA) and forms densely stained bands on mitotic chromosomes or chromocenters in interphase nuclei [13]. In plants with monocentric chromosomes and small genomes, this heterochromatin is usually confined to centromeric and pericentric regions [49]. In species with larger genomes, however, it can be found in additional subtelomeric and interstitial chromosomal loci [12], whereas plants with holocentric chromosomes usually lack distinguishable heterochromatic bands [19]. Heterochromatin is supposed to play an important role in chromosome segregation, gene regulation and the maintenance of genome stability [49], yet the processes shaping its distribution throughout the genome, and the role of underlying repetitive sequences, remain poorly understood [13]. This is in part due to our limited knowledge of the long-range sequence arrangement of repeat-rich heterochromatic regions which are in principle difficult to assemble [38].

SatDNA is organized in the genome in long arrays of almost identical, tandemly arranged units called monomers. Monomer sequences are typically hundreds of nucleotides long [27], although they can be as short as simple sequence repeats (<10 bp) [19] or reach over 5 kb [15]. Since monomer arrays can extend megabases in length, they present a significant challenge for even the most advanced genome assembly projects. Consequently, sequence composition of plant heterochromatin is traditionally elucidated by mapping repeats to heterochromatic chromosome bands using fluorescence *in situ* hybridization (FISH) [21]. However, this approach requires prior knowledge of the repeated sequences to be used as FISH probes. Despite the recent introduction of bioinformatic tools designed to retrieve satellite DNA sequences from short next generation sequencing (NGS) reads [34], [41], this reverse approach does not ensure that all repeats present in heterochromatic regions are revealed. Moreover, FISH-based methods have relatively limited resolution and are unable to reveal details of the internal structure of highly repetitive regions.

It has recently been demonstrated that repeat-rich genome regions, such as centromeres, can be efficiently assembled using long-read sequencing technologies that include the Pacific Biosciences and Oxford Nanopore platforms [26], [30]. The latter platform can generate “ultra-long” reads of up to 1 Mb [8] allowing for investigation of the long-range organization of genomic loci made of satellite DNA. In addition to greatly improving genome assembly [38], unassembled nanopore reads can also be utilized to examine the properties of satellite repeat arrays using dedicated bioinformatic tools [50].

**Fig. 1:**
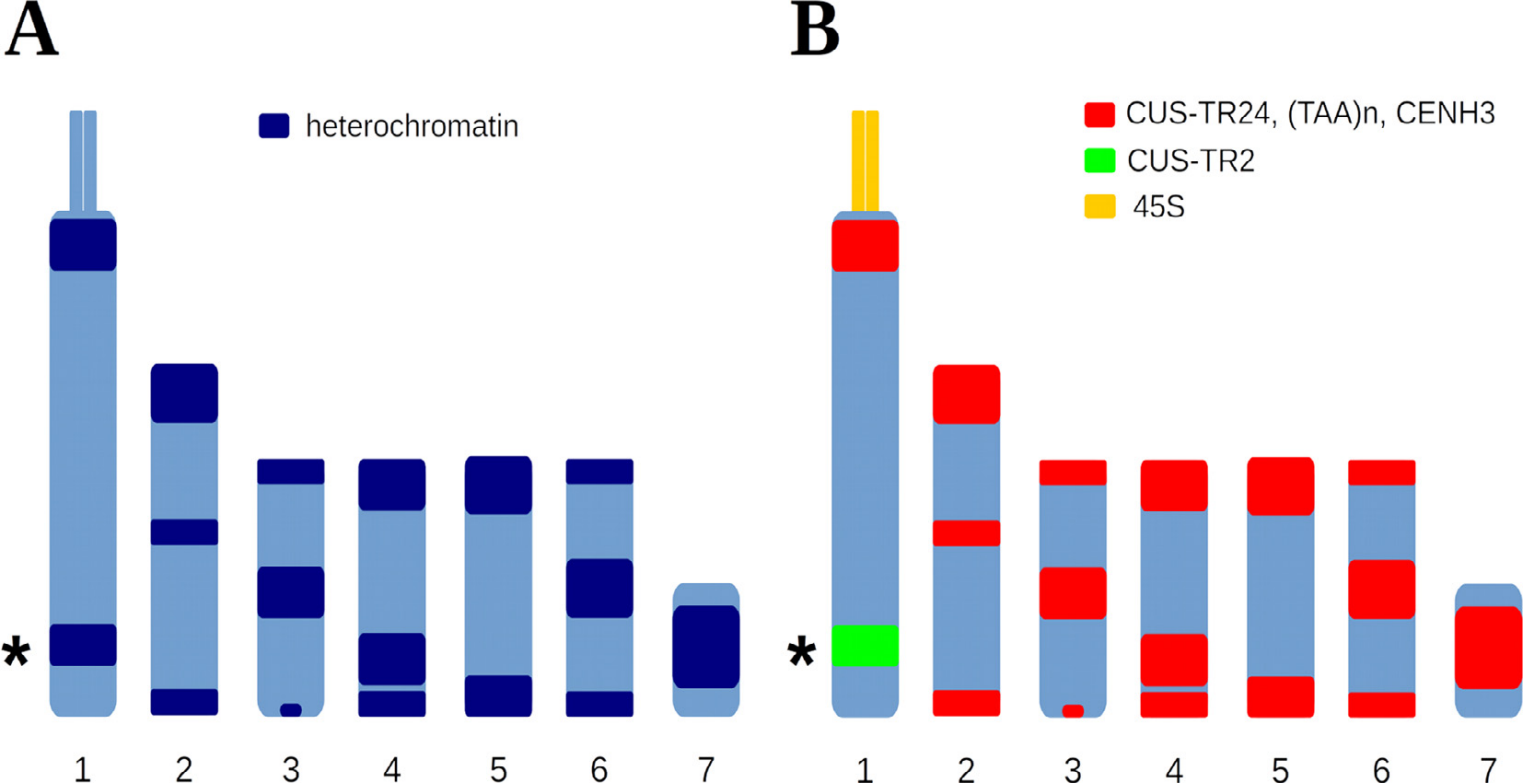
Schematic representation of the *Cuscuta europaea* karyotype (n = 7) with distribution of DAPI-positive heterochromatin bands (A) and tandem repeats (B). The loci containing CUS-TR24 repeats are associated with the CENH3 protein. The band on chromosome 1 that lacks CUS-TR24 but is composed of the satellite CUS-TR2 is marked with the asterisk.

Adapted from [36]

One of the most interesting satellite-rich heterochromatic genome regions has recently been described in the holocentric plant *Cuscuta europaea* [36]. Mitotic chromosomes of this species display large 4′,6-diamidino-2-phenylindole (DAPI)-positive heterochromatic bands (schematically depicted in Fig. 1), which are atypical for holocentric plants. Moreover, most of these heterochromatic bands are unique in their association with CENH3, a specific variant of canonical histone H3 that usually marks the position of active centromeres [44]. *C. europaea* CENH3 may have lost this function, however, as the mitotic spindle is able to attach to chromosomes at CENH3-free sites in this species [36]. The mechanism driving CENH3 deposition at heterochromatic bands in this species is currently unknown.

We have shown previously that heterochromatic bands on *C. europaea* chromosomes consist of 389 bp CUS-TR24 satellite repeats amplified to approximately 466,000 copies, accounting for 15.5% of the genome [33], [36]. FISH mapping of other *C. europaea* tandem repeats showed that heterochromatic regions also accumulated the simple sequence repeat (SSR) (TAA)n. Moreover, bioinformatic analysis of low-pass shotgun sequencing reads using the RepeatExplorer pipeline showed that the CUS-TR24 satellite can be interspersed with additional repeats [33]. Taken together, these findings indicated that the structure of *C. europaea* heterochromatic genome regions is complex.

In the present work, we have used ultra-long read sequencing to investigate the internal structure of the heterochromatic regions of *C. europaea* chromosomes. We adopted an assembly-free strategy, developed for the characterization of satDNA in the repeat-rich genome of *Lathyrus sativus* [50], for the genome-wide characterization of satellite arrays. This strategy employed a custom-made reference database for the identification of satellite arrays in individual nanopore reads. Nanopore reads representing significant genome coverage were then analyzed, revealing the prevalent length of arrays in the genome, sequence variations, and patterns of interspersion with other repetitive elements.

## 2 Material and methods

### 2.1 Genomic DNA isolation and nanopore sequencing

Seeds of *Cuscusta europaea* (serial number: 0101147) were obtained from the Royal Botanic Garden (Ardingly, UK). The plants were cultivated in the greenhouse and propagated vegetatively, using *Urtica dioica* as their host. High molecular weight nuclear DNA was isolated from young shoots of *C. europaea* employing the protocol described previously [50]. Five grams of tissue was frozen in liquid nitrogen, ground to a fine powder and incubated for 5 min in 35 ml ice-cold H buffer (1 × HB, 0.5 M sucrose, 1 mM phenylmethyl-sulphonylfluoride (PMSF), 0.5% (v/v) Triton X-100, 0.1% (v/v) 2-mercaptoethanol) prepared fresh from a 10 × HB stock (0.1 M TRIS-HCl pH 9.4, 0.8 M KCl, 0.1 M EDTA, 40 mM spermidine, 10 mM spermine). The homogenate was filtered through 48 μm nylon mesh, adjusted to 35 ml with 1 × H buffer, and centrifuged at 230 × g for 15 min at 4°C. The pelleted nuclei were resuspended in 35 ml H buffer, centrifuged at 230 × g for 15 min at 4°C, and the resulting pellet was resuspended in 15 ml TC buffer (50 mM TRIS-HCl pH 7.5, 75 mM NaCl, 6 mM MgCl_2_, 0.1 mM CaCl_2_). A final centrifugation was performed at 400 × g for 5 min, and the nuclei were resuspended in 2 ml TC. The suspension of nuclei was mixed with an equal volume of 2 × CTAB buffer (1.4 M NaCl, 100 mM Tris-HCl pH 8.0, 2% CTAB, 20 mM EDTA, 0.5% (w/v) Na_2_S_2_O_5_, 2% (v/v) 2-mercaptoethanol) and incubated at 50°C for 30–40 min. The solution was extracted with chloroform: isoamylalcohol (24:1) using MaXtract^TM^ High Density Tubes (Qiagen) and precipitated with a 0.7 × volume of isopropanol using a sterile glass rod to collect the DNA. Following two washes in 70% ethanol, the DNA was dissolved in TE and treated with 2 μl RNase Cocktail^TM^ Enzyme Mix (Thermo Fisher Scientific) for 1 h at 37°C. Finally, the DNA was further purified by mixing the sample with a 0.5 × volume of CU and a 0.5 × volume of IR solution from the Qiagen DNeasy PowerClean Pro Clean Up Kit (Qiagen), centrifugation for 2 min at 15,000 rpm at room temperature and DNA precipitation from the supernatant using a 2.5 × volume of 96% ethanol. The DNA was dissolved in 10 mM TRIS-HCl pH 8.5 and stored at 4°C.

Sequencing libraries were prepared from 3 μg of the purified, partially fragmented DNA (from ~20 kb to >100 kb) using a Ligation Sequencing Kit SQK-LSK109 (Oxford Nanopore Technologies), following the manufacturer’s protocol. Briefly, the DNA was treated with 2 μl NEBNext FFPE DNA Repair Mix and 3 μl NEBNext Ultra II End-prep enzyme mix in a 60 μl volume that included 3.5 μl FFPE and 3.5 μl End-prep reaction buffers (New England Biolabs). The reaction was performed at 20°C for 5 min and 65°C for 5 min. Subsequently, the DNA was purified using a 0.4 × volume of AMPure XP beads (Beckman Coulter); because long DNA fragments caused clumping of the beads and were difficult to detach, elution was performed with 5 mM TRIS-HCl (pH 8.5) for 40 min. Subsequent steps, including adapter ligation using NEBNext Quick T4 DNA Ligase and library preparation for sequencing, were performed as recommended. The whole library was loaded onto a MinION FLO-MIN106 R9.4.1 flow cell and sequenced until the number of active pores dropped below 40 (19–20 h). Two independent sequencing runs were performed, and the resulting raw reads were deposited into the European Nucleotide Archive (https://www.ebi.ac.uk/ena) under run accession numbers ERR5237073 and ERR5237074.

### 2.2 Bioinformatic analysis of nanopore reads

Raw nanopore reads were basecalled using the Oxford Nanopore basecaller Albacore (ver. 2.3.4). Quality-filtering of the resulting FastQ reads, and their conversion to the FASTA format, was performed with BBDuk (part of the BBTools, https://jgi.doe.gov/data-and-tools/bbtools/) run with the parameter maq = 8. Reads shorter than 30 kb were discarded. Unless stated otherwise, all bioinformatic analyses were implemented using custom Python and R scripts, and executed on a Linux-based server equipped with 64 GB RAM and 32 CPUs.

Self-similarity dot-plot analysis of individual nanopore reads was done using the Gepard [23] and Dotter programs [46], and the annotated dot-plots used for the figures were generated using FlexiDot [45]. Repeat annotation in nanopore reads and subsequent analysis of the length distribution of tandem repeat arrays and their interspersion with other repetitive sequences followed the procedures described previously [50]. Briefly, the repeats were identified and annotated in the nanopore reads based on their similarities to a custom-made reference database. The database included consensus sequences that were representative of all major repeat groups identified in the *C. europaea* genome using the RepeatExplorer analysis of Illumina reads [33]. For each family of tandem repeats and LINE elements, the reference sequences in the database were placed in the same orientation to allow for the evaluation of their mutual orientations in the nanopore reads. Sequence similarities were detected using LASTZ [18]. The search parameters and processing of the resulting similarity hits were as described previously [50]. The reference database and custom scripts used for the analysis are available from GitHub (https://github.com/vondrakt/nanopore-read-annotation-Cuscuta-europaea.git).

### 2.3 Analysis of LINE sequences

Consensus sequences of full-length LINE elements were reconstructed from contigs produced by the RepeatExplorer [33]. The positions of regions coding for retrotransposon proteins in these sequences were detected by DANTE (https://repeatexplorer-elixir.cerit-sc.cz/) based on their similarity to the REXdb protein database [32]. Phylogenetic analysis of LINEs was performed using reverse transcriptase (RT) protein domain sequences extracted by DANTE from *C. europaea* contigs. These sequences were supplemented with a set of 71 randomly selected reference RT domains representing different lineages of plant LINEs from Eudicot plant species [20]. Multiple sequence alignment of RT sequences was done using the Muscle alignment program [11] and refined by manual inspection using Seaview [16]. A Neighbor-Joining phylogenetic tree was calculated using Geneious Prime 2020.1.1 (https://www.geneious.com) with default parameters.

Associations of the individual LINE lineages with CUS-TR24 repeats were investigated by extracting all identified LINE sequences from the nanopore reads and dividing them into two groups based on their presence in a 10 kb region adjacent to CUS-TR24 arrays. The elements located within these regions were labeled as associated while those located >10 kb from CUS-TR24 were classified as not associated. Sequences from both groups were assigned to a LINE lineage using the LASTZ program, which compared each sequence with a set of full-length reference LINE sequences. To obtain a unique hit for each sequence, the best hit for each sequence was identified based on the highest bitscore. The LASTZ command for running the alignment was ‘lastz query[multiple,unmask] database –format = general:name1,size1,start1,length1,strand1,name2,size2,start2,length2,strand2,identity,score --ambiguous = iupac --xdrop = 10 –hspthresh = 1000′.

Insertion sites of LINEs were mapped to a dimer of CUS-TR24 consensus sequence for full-length (5–7 kb) LINE elements. A 200 bp window was extracted from each side of the LINE. Windows that were shorter than 190 bp or that had <190 bp annotated as CUS-TR24 were discarded. These windows were then aligned to the CUS-TR24 dimer using the LASTZ alignment program and the command described above. The alignment was filtered by bitscore so that each window had a unique hit to the CUS-TR24 dimer, and the insertion sites were recorded. The insertion site frequencies from the identical parts of CUS-TR24 the dimer were merged to produce a monomer insertion site profile.

### 2.4 Chromosome preparation and FISH

Mitotic chromosomes for FISH experiments were prepared from shoot apical meristems fixed in a 3:1 solution of methanol: glacial acetic acid for at least 24 h, without previous treatment. The fixed meristems were washed three times in distilled water for 5 min. To remove the cell wall, washed meristems were incubated in a solution of 2% cellulase and 2% pectinase in PBS for 70 min at 37°C, followed by two washes with cold distilled water. Slides were prepared using the flame-drying method; meristems were macerated in a drop of cold 3:1 ethanol: glacial acetic acid fixative solution using fine-pointed forceps on a glass slide, which was subsequently warmed over an alcohol flame and air-dried before immediate use or storage at 4°C. An oligonucleotide probe for CUS-TR24 (5′-AGT GTC ACA AAT ACT TAG CCT TAT CTC TAT GAT TTA GCG TTT TCA GCG AA-3′) was labeled with fluorescein isothiocyanate (FITC) at its 5′ ends during synthesis (Integrated DNA Technologies, Leuven, Belgium). Fragments of other probes were PCR-amplified from genomic DNA of *C. europaea* and cloned into pCR4-TOPO vector (Thermo Fisher Scientific). PCR primer sequences were 5′-CCT CTT TGA TAT TGG AGA TAA TAA ATC-3′ and 5′-GGC AAG GTC ATA ATC AGC A-3′ for L1-CS_cl3, 5′-GTT TGA TAT TGG GGA TGA CAA-3′ and 5′-AAC ACC TCC CAA GAA AAT ATT AGA T-3′ for L1-CS_cl48, and 5′-AGG CAG ATC TTC CGA GGT A-3′ and 5′-AAA GTC AAG CAC AAG CAT CC-3′ for the RTE probe; the sequences of the cloned probes are available from GenBank under accession numbers MN625503, MN625506 and MN625501, respectively. These probes were labeled with biotin-16-dUTP (Roche, Mannheim, Germany) using nick translation [22]. FISH was performed as described previously [28] with a hybridization and washing temperature of 37°C. Slides were counterstained with DAPI, mounted in Vectashield mounting medium (Vector Laboratories, Burlingame, CA, USA), and examined using a Zeiss AxioImager.Z2 microscope with an Axiocam 506 mono camera. Images were captured and processed using ZEN pro 2012 software (Carl Zeiss GmbH).

## 3 Results

### 3.1 Nanopore sequencing and initial analysis of the reads provides the first insight into the complex structure of the CUS-TR24 loci

The sequencing of high molecular weight nuclear DNA from *C. europaea* was performed on the Oxford Nanopore MinION device using a 1D ligation sequencing kit. Quality-filtered reads were pooled from two independent sequencing runs and filtered for a minimum length of 30 kb, resulting in the selection of 96,528 reads for further analysis. Selected reads were up to 239 kb in length (N50 = 56.9 kb) and represented 5.9 Gbp of sequence data (5-fold coverage of 1169 Gb/1C *C. europaea* genome [33]).

**Fig. 2:**
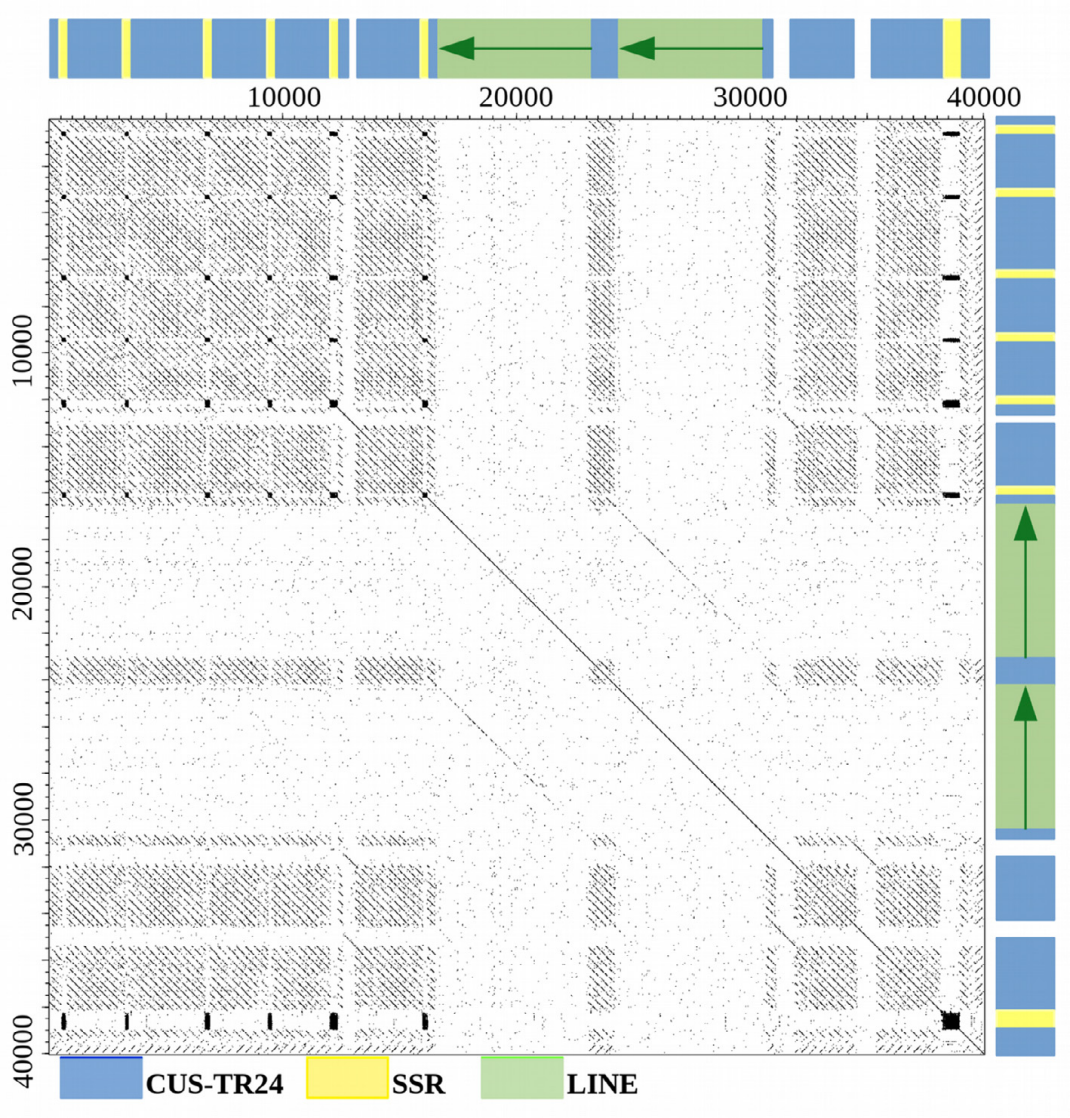
Sequence organization of CUS-TR24 loci revealed by self-similarity dot-plot analysis of individual nanopore reads. A typical sequence arrangement is demonstrated here on a dot-plot from a 40 kb portion of a 98 kb read. Sequence annotation within the read is provided along the dot-plot axes, with colored rectangles representing CUS-TR24 satellite arrays (blue), SSRs (yellow) and LINEs (green, with the arrow showing the 5′→3′ orientation). Dot-plot of the entire read and additional dot-plot examples are provided in Supplementary Fig. S1. (For interpretation of the references to color in this figure legend, the reader is referred to the web version of this article.)

Initial sequence analysis of randomly selected reads containing CUS-TR24 sequences was performed using self-similarity dot-plots to investigate their internal structure (Fig. 2). The dot-plots revealed that these reads had a complex and variable structure composed of arrays of tandemly repeated CUS-TR24 monomers frequently interrupted with short regions of simple sequence repeats (SSRs). These SSRs were mostly (TAA)n motifs, confirming our previous finding, from FISH experiments, that (TAA)n repeats co-localize with CUS-TR24 [36]. In addition, other, less frequent motifs were detected, including a (TGA)n motif and irregular repetitions of SSR-like sequences. It was also evident from the dot-plots that CUS-TR24 arrays are often interrupted with common sequences identified as fragments of mobile elements with multiple copies found within and between reads (Fig. 2). Structure of these elements and sequences of their open reading frames coding for reverse transcriptase and endonuclease proteins led to their classification as LINE retrotransposons.

### 3.2 Computational analysis of all nanopore reads reveals a general pattern of sequence arrangement in CUS-TR24-containing heterochromatin

To investigate if the patterns uncovered by the dot-plot analysis of selected reads represented general features of the genomic loci containing CUS-TR24 repeats, we performed a computational analysis of their properties across the whole set of nanopore reads. A reference database containing a representative set of CUS-TR24, SSRs and LINE sequences was assembled and used to identify regions containing these repeats in individual nanopore reads. The lengths of these regions and their mutual interspersion were then evaluated. The reference database was also supplemented with other *C. europaea* tandem repeats including the abundant CUS-TR2 satellite and 45S rDNA sequences, and by representatives of major groups of mobile elements that were previously characterized from the *C. europaea* genome [33].

**Fig. 3:**
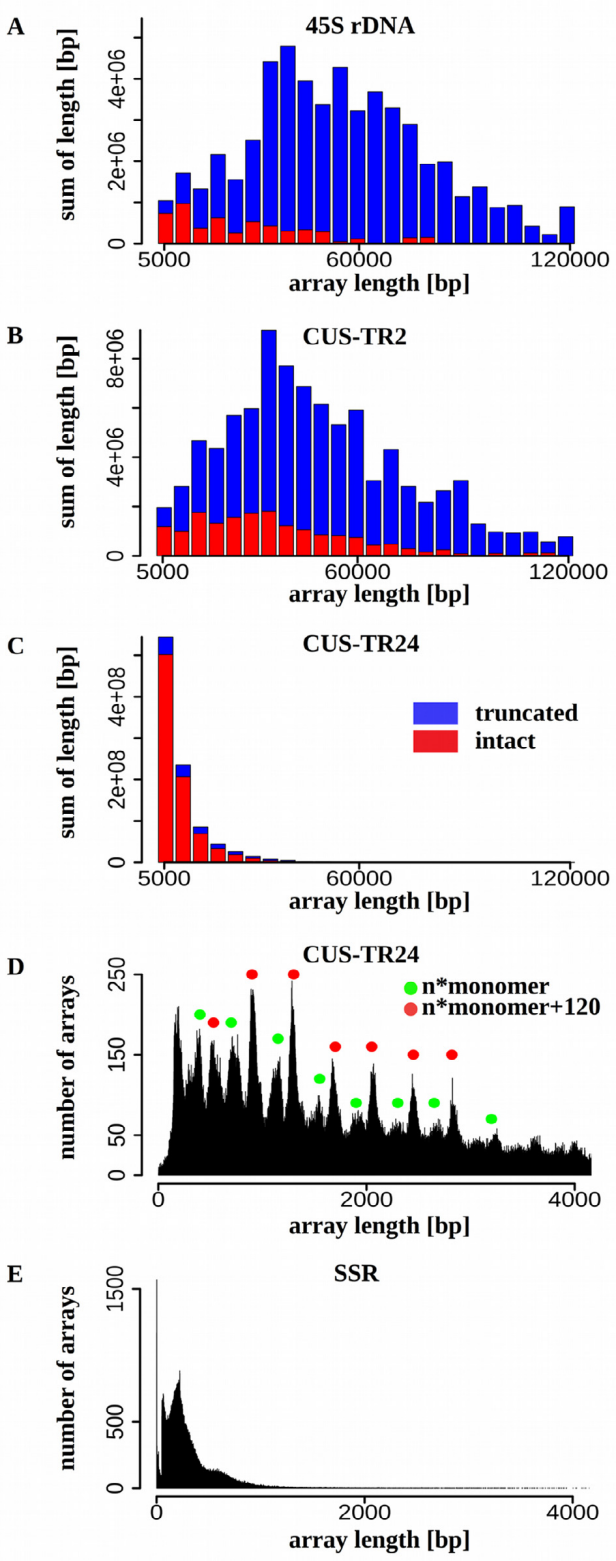
Length distribution of the satellite repeat arrays. (A-C) The lengths of the arrays detected in nanopore reads are displayed as weighted histograms with a bin size of 5 kb; the last bin includes all arrays longer than 120 kb. Arrays completely embedded within a read (red bars) are distinguished from truncated arrays positioned at the end of a read (blue bars). Due to array truncation, the latter values are underestimation of the lengths of corresponding genomic arrays and should be considered as lower bounds of the respective array lengths. (D-E) The distribution of CUS-TR24 and SSR array length plotted in 1 bp resolution. The formulas provided in (D) explain the prevalent array lengths represented by the peaks marked with corresponding colors. (For interpretation of the references to color in this figure legend, the reader is referred to the web version of this article.)

The analysis of the length distribution of tandem repeat arrays revealed remarkable differences between the investigated repeats. The array length distributions were visualized as weighted histograms with a bin size of 5 kb, distinguishing complete and truncated satellite arrays (Fig. 3). While 45S rDNA and CUS-TR2 sequences were almost exclusively present as long contiguous arrays of up to hundreds of kilobases that extended beyond the lengths of most reads (Fig. 3A,B), CUS-TR24 arrays were much shorter, with over 96% of them not exceeding 10 kb (Fig. 3C). A detailed plot of CUS-TR24 array length distribution showed a series of peaks ranging from ~200 bp to 4 kb (Fig. 3D). The occurrence of these peaks and their spacing suggested that the CUS-TR24 arrays are not terminated at random positions but instead differ by multiples of the consensus monomer length (389 bp). The observed pattern of prominent peaks interlaced by smaller ones could then be explained by the presence of two variants of array termination in the genome: the prominent peaks represented arrays containing multiple complete monomers terminated by a truncated monomer sequence of ~120 bp, while a series of smaller peaks corresponded to multiples of full-length monomers (Fig. 3D). The size distribution of SSR arrays (Fig. 3E) did not show any regular pattern and were mostly of a short length (<400 bp).

**Fig. 4:**
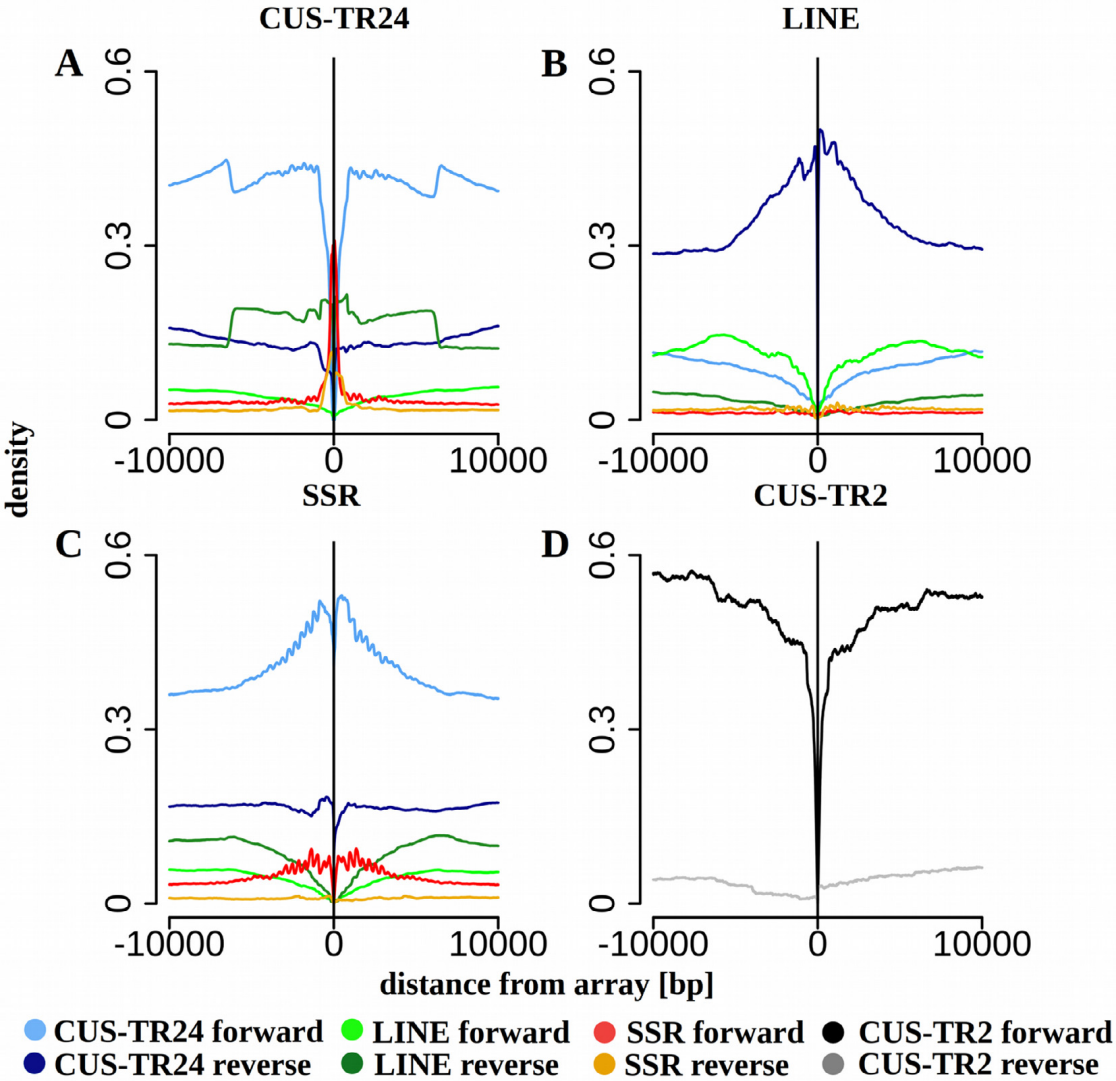
Sequence composition of genomic regions adjacent to CUS-TR24 arrays (A), LINE elements (B), SSRs (C) and CUS-TR2 arrays (D). The plots show the proportions of repetitive sequences identified within 10 kb regions upstream (positions −1 to −10,000) and downstream (1 to 10,000) of the arrays of tandem repeats (A, C, D) or insertion of LINE elements (B). The vertical line shows the array or LINE position, and the plots are relative to the forward-oriented sequences. Only the repeats detected in proportions exceeding 0.05 are plotted (colored lines).

Next, we investigated patterns of interspersion of CUS-TR24 sequences with other repeats by examining the presence and orientation of repeats within 10 kb regions directly adjacent to each CUS-TR24 array. Results were pooled from all reads, and the frequencies at which different repeats were associated with CUS-TR24 arrays were summarized (Fig. 4A). This analysis revealed that about 40% of CUS-TR24 arrays are terminated by short SSR repeats (30% in forward and 10% in reverse orientation with respect to the CUS-TR24 arrays). However, their broader neighborhood (1–10 kb) was most frequently (40–45%) occupied by another CUS-TR24 array in the same orientation, while CUS-TR24 sequences in the opposite orientation were less frequent (10–15%). Up to 20% of CUS-TR24 arrays were directly adjacent to LINE elements, with the LINE elements frequently in reverse orientation to the CUS-TR24 consensus. Similar analysis of LINE elements revealed that up to 50% of the genome regions directly adjacent to LINE sequences consisted of CUS-TR24 in a reverse orientation (Fig. 4B). SSR arrays were found to be similarly surrounded by CUS-TR24 sequences and, to a lesser extent, by further SSR sequences (Fig. 4C). The distinct peaks evident in the CUS-TR24 and SSR density plots reflect the interlaced pattern of these repeats, with SSRs separated by CUS-TR24 arrays of various lengths that differ by multiples of CUS-TR24 monomer size (Fig. 4C). In contrast to CUS-TR24, another highly amplified satellite, CUS-TR2, did not show preferential association with other repetitive sequences (Fig. 4D), consistent with the observation that this satellite usually forms long, homogeneous arrays (Fig. 3B).

### 3.3 CUS-TR24 sequences are interspersed with a specific lineage of LINEs due to its insertional target site preference

**Fig. 5:**
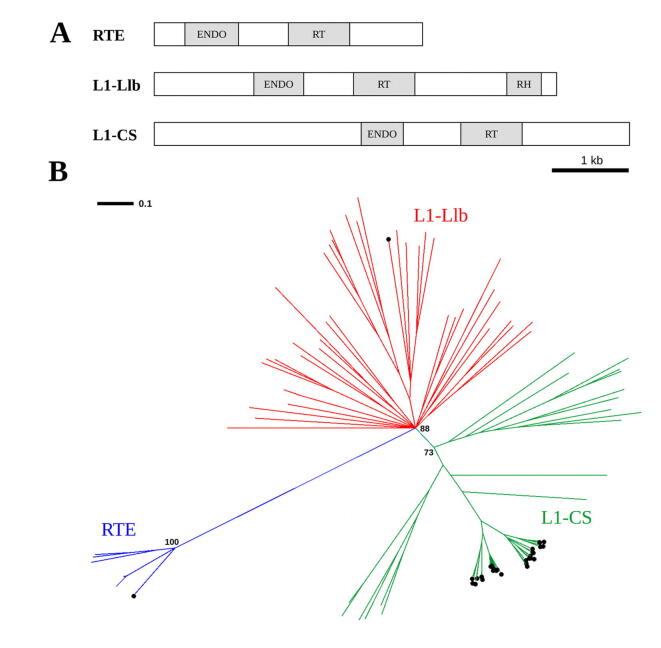
Structure of the reconstructed consensus sequences representing three distinct LINE element groups identified in the *C. europaea* genome (A). Groups were assigned to phylogenetic lineages defined by Heitkam et al. [20] according to similarities of their RT domain sequences (B). The branches of the neighbor-joining tree labeled with circles represent RT sequences extracted from *C. europaea* elements. The remaining branches represent reference sequences collected from various plant species [20]. Bootstrap values are provided for the major nodes and the scale bar indicates numbers of changes per site.

The observed association of LINEs with CUS-TR24 arrays prompted us to perform detailed characterization of these sequences in the *C. europaea* genome. Using previously published data on repeat variation in *Cuscuta* [33], we defined three major LINE element groups in the *C. europaea* genome. These groups corresponded to sequence clusters or super-clusters generated by the similarity-based repeat clustering of Illumina reads employing the RepeatExplorer pipeline [35]. Representative full-length elements were reconstructed for each group using consensus sequences produced by the RepeatExplorer. The structure of these elements is provided in Fig. 5A, showing positions of the regions coding for reverse transcriptase (RT), RNase-H (RH), and endonuclease (ENDO) protein domains. We used protein sequences obtained by conceptual translations of the RT-coding regions to assign the identified elements to the phylogenetic lineages of plant LINEs defined by Heitkam et al. [20]. A Neighbor-Joining tree constructed from multiple alignment of RT sequences sampled from various plant species [20] revealed that the three groups of *C. europaea* LINEs belong to the three major branches of the tree, representing L1 LINE-CS (L1-CS), L1-Llb, and RTE lineages (Fig. 5B). Repeat clustering data estimated the proportions of these lineages in the *C. europaea* genome to be 4.26%, 0.08%, and 0.45%, respectively.

**Fig. 6:**
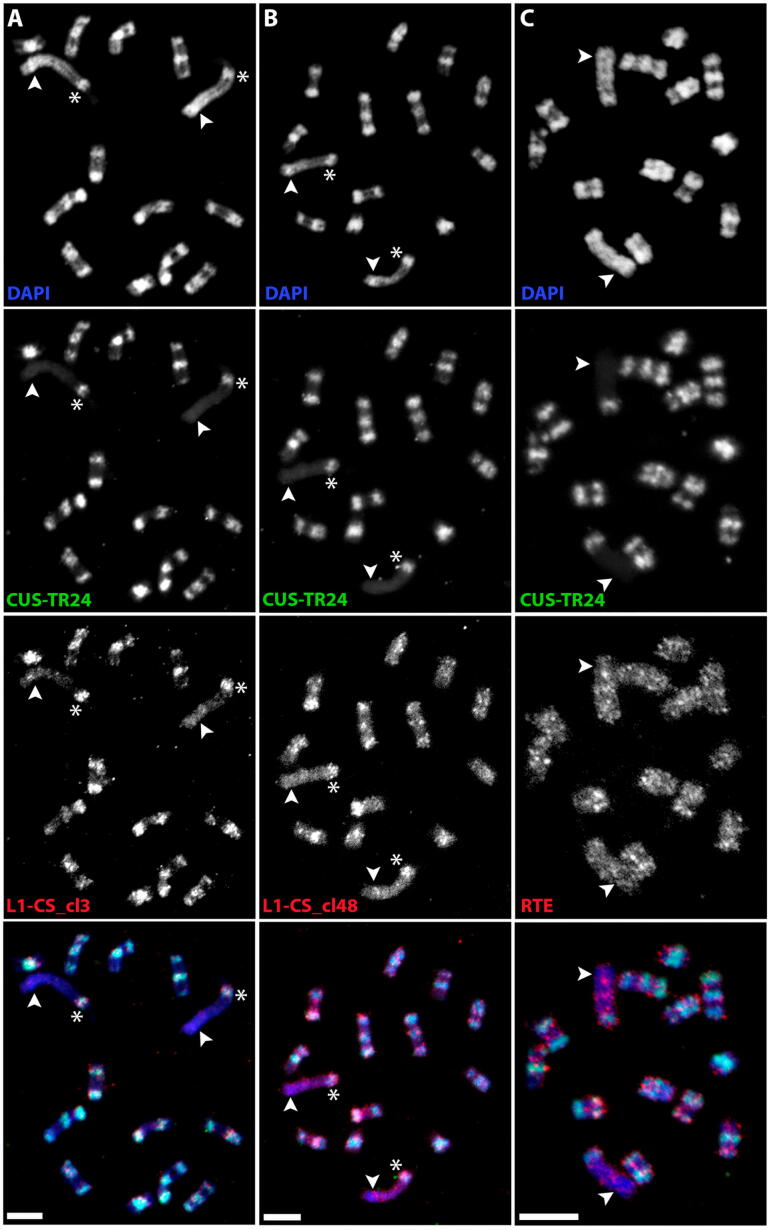
Distribution of LINE sequences on metaphase chromosomes of *C. europaea*. Two-color FISH experiments were performed to detect LINEs (red channel) and CUS-TR24 sequences (green). The chromosomes were counterstained with DAPI (blue). Individual channels and corresponding merged color images are shown for experiments including LINE probes L1-CS_cl3 (A), L1-CS_cl48 (B) and RTE (C). Arrowheads mark the position of DAPI-positive heterochromatic band on the chromosome 1 that lacks CUS-TR24 repeats (for comparison, the CUS-TR24-containing band on the same chromosome that is also enriched for L1-CS LINEs is marked with asterisk). See also Fig. 1 for a schematic of this karyotype. Bar = 5 μm. (For interpretation of the references to color in this figure legend, the reader is referred to the web version of this article.)

We re-analyzed the nanopore read data taking the classification of LINE element by lineage into account, examining LINE sequences located in proximity (up to 10 kb) to CUS-TR24 arrays and comparing them with all remaining LINE elements detected in the nanopore reads. This analysis revealed that 91% of L1-CS elements were associated with CUS-TR24 arrays, while the other two lineages showed no such strong association (Table 1). To verify these results, we designed hybridization probes for L1-CS and RTE sequences and visualized their distribution on metaphase chromosomes of *C. europaea* using FISH (the L1-Llb elements were not examined due to their low proportion in the genome). Two different probes were used for L1-CS to account for sequence variation among these elements. The FISH signals of both probes were much stronger in the DAPI-positive heterochromatic bands than in the euchromatic chromosome regions (Fig. 6A,B). In addition, only bands known to contain CUS-TR24 repeats were strongly labeled, while a band on chromosome 1 consisting of CUS-TR2 (Fig. 1) lacked these strong FISH signals. Conversely, the RTE probe generated labeling patterns that were uniformly scattered along whole chromosomes (Fig. 6C), suggesting that these elements are evenly dispersed in the genome. These experiments thus confirmed that CUS-TR24 loci are specifically enriched with LINEs of the L1-CS lineage.

**Table 1:** Estimated proportions of LINE elements associated with CUS-TR24 repeats.

Lineage	Associated with CUS-TR24		Elements
	YES	NO	scored
L1-CS	91%	9%	97,860
L1-Llb	32%	68%	7302
RTE	14%	86%	21,645

**Fig. 7:**
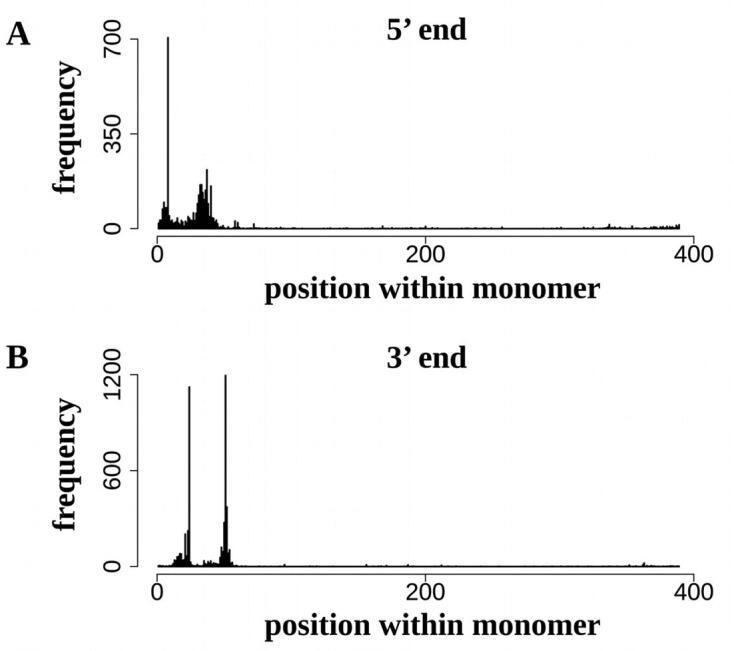
Analysis of the insertional target sites of LINE elements within CUS-TR24 monomers. Plots show the frequency of 5′ (A) and 3′ ends (B) of LINEs mapped to individual positions along a CUS-TR24 consensus monomer (the monomer sequence is provided in Fig. 8A). Due to target site duplication generated upon element insertion, the mapped positions of 5′ and 3′ ends are shifted by approximately 13–16 bp. An example of CUS-TR24 sequence with LINE insertion is provided in Supplementary Fig. S3, including also target site duplication generated upon LINE insertion.

**Fig. 8:**
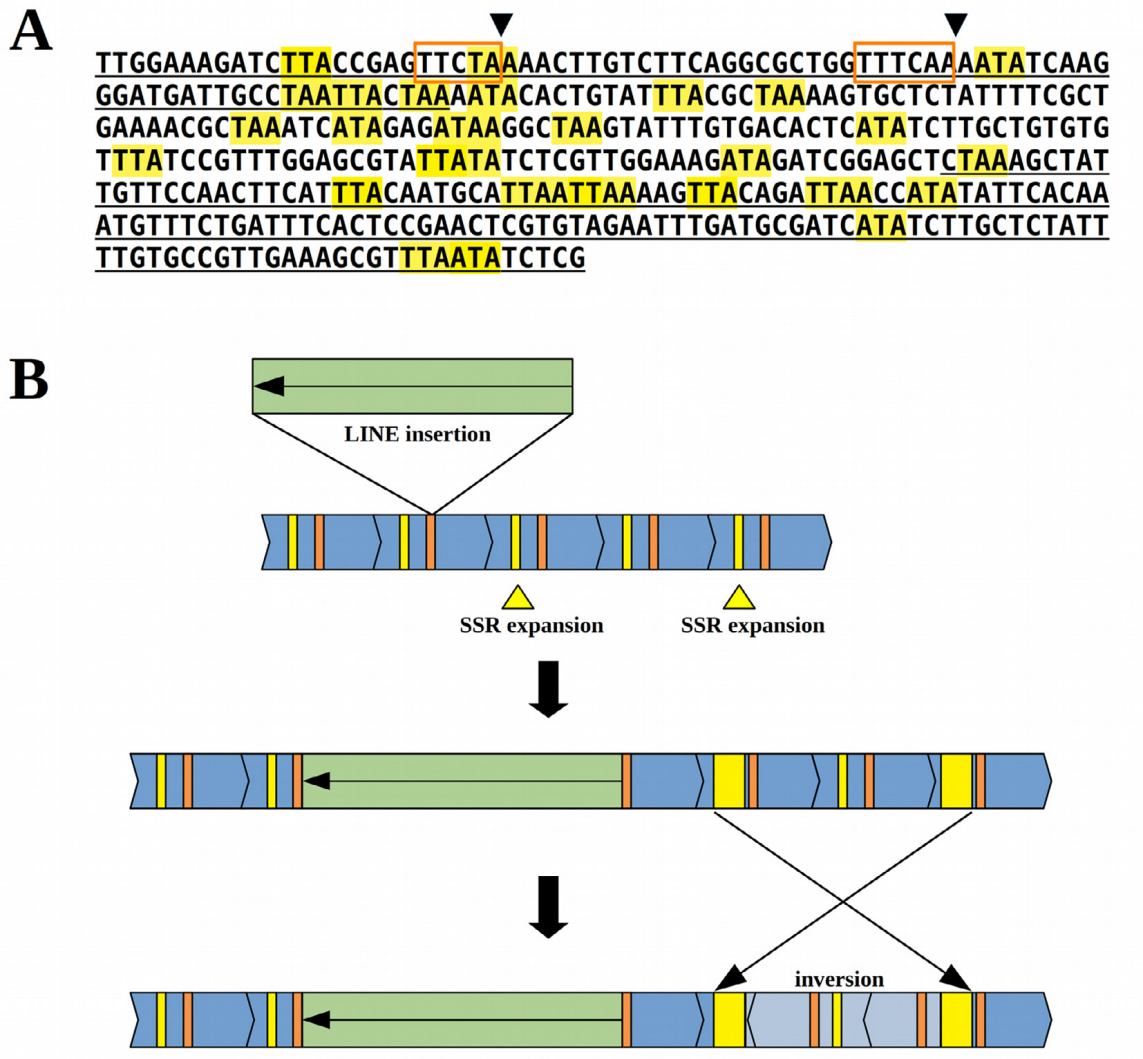
(A) Consensus sequence of a CUS-TR24 monomer. The target sequences for LINE insertion are marked with orange rectangles (the putative cleavage sites are marked with arrowheads). The (TAA) motifs and their variants are highlighted in yellow, and the monomer region frequently lost at CUS-TR24/SSR junctions is underlined. (B) A model to represent the processes leading to the complex structure of CUS-TR24 loci. The ancestral CUS-TR24 monomer arrays (blue) contain hotspots for SSR emergence from (TAA) motifs (yellow) and LINE target sites (orange). These arrays become fragmented by concurrent SSR expansion and insertion of new LINE elements, and undergo further rearrangements, including segmental duplications and inversions. (For interpretation of the references to color in this figure legend, the reader is referred to the web version of this article.)

The specific association of L1-CS elements with CUS-TR24 repeats prompted us to investigate if this association might result from an insertional preference for this LINE lineage. LINEs insert into the genome via target-site primed reverse transcription, generating target site duplication (TSD) upon their insertion [29]. Selective insertional targeting to specific sequence motifs has been described for some LINE families [7]. If this mechanism was also functional in the *C. europaea* L1-CS elements, it could explain the observed interspersion patterns, if the LINE target sequence was conserved in CUS-TR24 monomers. Indeed, the mapping of LINE insertions with respect to the CUS-TR24 consensus monomer showed clear preference for two adjacent regions of the monomer (Figs. 7 and 8A). These two insertion sites had consensus sequences of 5′-TTCTA-3′ and 5′-TTTCAA-3′, similar to the cannonical cleavage site of mammalian L1 elements (5′-TTTTAA-3′) [47].

### 3.4 The model for the origin of the complex structure of CUS-TR24 loci

Taken together, our findings indicated that the complex sequence arrangement of heterochromatic loci containing CUS-TR24 repeats resulted from a combined action of several processes, outlined in Fig. 8B. It appears that the nucleotide sequence of the CUS-TR24 monomer played a crucial role in these processes by providing target sequences for the L1-CS element insertions and hotspots for the emergence of SSR arrays (Fig. 8A). In the proposed model, we presume that ancestral arrays of CUS-TR24 were amplified in the *C. europaea* genome (Fig. 8B). The frequent occurrence of (TAA) motifs within the monomer sequences (highlighted in Fig. 8A) provided a template for their occasional conversion and/or expansion into SSR arrays, possibly via the replication strand slippage mechanism known to generate microsatellite sequences [43]. The AT-rich sequences may also constitute fragile sites that are prone to DNA breakage and structural rearrangements [2]. Our detailed inspection of the CUS-TR24/SSR boundaries in multiple nanopore reads revealed that the presence of expanded (TAA)n motifs within CUS-TR24 arrays was frequently associated with the truncation of neighboring monomer sequences (Fig. 8A). The length of truncated monomers varied between ~120–150 bp, which roughly corresponds to the observed size distribution pattern of CUS-TR24 arrays (Fig. 3D), consisting of multiple full-length monomers terminated by the truncated monomer sequence of ~120 bp.

Concurrent with the emergence of SSRs, the CUS-TR24 monomers were specifically targeted by L1-CS lineage LINEs (Fig. 8B). Since these CUS-TR24-associated LINEs are relatively heterogeneous in their nucleotide sequences (Supplementary Fig. S2 and Fig. 2), it is likely that they originated from the retrotransposition of multiple master elements. Finally, the CUS-TR24 loci were probably shaped by additional processes including segmental duplications, inversions (both are evident from the dot-plot analysis; Supplementary Fig. S1), and possibly recombination-based deletions, resulting in the present complex structure of these loci.

## 4 Discussion

In this study, we uncovered the complex sequence structure of genomic loci containing the satellite CUS-TR24, which constitute most of the heterochromatic bands on holocentric chromosomes of *C. europaea*. Satellite DNA is known to be the major component of constitutive heterochromatin in eukaryotic genomes [14], [40], being supposedly arranged in long contiguous arrays that are only sparsely interrupted by random insertions of mobile elements. Such arrangement has been documented for some human and plant satellites [25], [30], [50], and it has also been found for the other abundant satellite family in *C. europaea*, CUS-TR2. This contrasts with the genome organization of the CUS-TR24 repeats, which are highly fragmented due to their interspersion with short SSR arrays and insertions of LINE elements. Such a complex structure was unexpected for highly amplified satellite repeat, especially considering that its amplification in *C. europaea* occurred relatively recently as judged from the absence of the CUS-TR24 repeats from closely related species *C. epithymum* [33]. On the other hand, arrays of abundant satellite repeats in maize were found to be highly fragmented by retrotransposon insertions [26]. Mobile elements were also proposed to generate complex arrangements and even facilitate genomic dispersal of satellite repeats in other species [37], [42], [50]. Since detailed studies of satellite repeat arrays are still scarce, it is yet to be elucidated what is the prevailing type of satDNA organization and how it is affected by various factors like the age of the arrays or their location in the genome.

To explain the origin of a complex pattern shared among CUS-TR24 loci, we considered two different scenarios, proposing that either (1) there was an ancestral, low-copy repeat composed of adjacent CUS-TR24, SSR, and LINE sequences that became amplified and spread throughout the genome as a new compound monomeric unit; or (2) the pattern resulted from ongoing and concurrent processes of CUS-TR24 amplification, the emergence of SSRs from proto-SSR units, and the insertional targeting of LINEs during their genomic proliferation.

The compound monomers proposed in the first scenario have already been described for several satellite repeats. For example, a 4.7 kb-long monomer of the *Sobo* satellite from *Solanum bulbocastanum* originated from part of an LTR-retrotransposon and a genomic tandem repeat [48]. Similar satellites with long monomers consisting of unrelated, repeated and/or low-copy genomic sequences have been described from *Solanum tuberosum* [15] and *Secale cereale* [24]. However, the monomer sequences of these satellites are highly homogenized throughout the genome, with up to 99% similarity between copies [48], and therefore the arrangement of the original sequence components is identical in all monomers. No such conserved arrangement of CUS-TR24, SSR, and LINE sequences occurs at CUS-TR24 loci, making it unlikely that they were amplified as a single conserved monomer unit. In addition, there is variation in the presence and length of the SSR arrays in CUS-TR24 monomers (Fig. 2 and Supplementary Fig. S1), and considerable LINE sequence diversity (Supplementary Fig. S2), which suggests that they do not originate from a single insertion into an ancestral satellite array. However, the association of heterogeneous LINE sequences with CUS-TR24 can be explained by recurrent retrotransposition of multiple template elements. Despite their sequence variation, these elements belong to the same phylogenetic lineage of LINEs and share insertional target sites (Fig. 7). Considering these facts, we favor the explanation provided by the second scenario, which was included into the proposed model of the evolution of CUS-TR24 loci (Fig. 8).

A notable feature of the CUS-TR24 loci is their association with the CENH3 protein [36] which serves as an epigenetic marker of active centromeres in all plant species studied so far [6], [44]. However, *C. europaea* CENH3 may have lost this function: the distribution of CENH3 on chromosomes does not correlate with the attachment of the mitotic spindle [36]. It is supposed that CENH3 deposition to plant centromeres is independent of the underlying centromeric repeats; instead, it is a part of an epigenetically determined self-propagation loop based on the interactions of CENH3 chaperones and the additional constitutive centromere-associated network (CCAN) proteins [17]. However, the mechanism driving CENH3 deposition in *C. europaea* is unknown. It is possible that there is a sequence-dependent interaction between CENH3 (or its chaperone) and CUS-TR24 repeats, in a manner similar to the interaction of human centromeric protein CENP-B with a 17 bp CENP-B-box sequence within centromeric alpha satellites [10]. However, such sequence-specific deposition of CENH3 has not been reported in any plant species.

Although repeat-rich regions of the genome are generally transcriptionally silent, it has been reported that transcriptional activity at centromeric repeats plays an important role in CENH3 deposition [9], [39]. In this respect, the accumulation of LINE elements in the CUS-TR24 loci may be of interest, as these elements could initiate transcription of adjacent sequences, which in turn may promote CENH3 deposition. In support of this hypothesis is the finding that LINE-L1 transcripts are an essential component of human neocentromeres [5]. LINEs represent major repeats associated with centromeric chromatin in *Drosophila* [4] and centromere-specific LINE elements have been reported in the sunflower genome [31]. Although we currently cannot provide an explanation for the observed co-localization of CENH3 with heterochromatin containing CUS-TR24 repeats, the findings discussed above warrant further investigation of kinetochore composition and centromere determination in the holocentric *Cuscuta* species.

This work provides evidence for a new type of highly complex sequence arrangement in plant constitutive heterochromatin. It also demonstrated the potential of long-read sequencing technologies to fill gaps in our knowledge of the satellite DNA-rich regions of eukaryotic genomes that are otherwise hard to investigate. Although the ultra-long sequence reads are mostly used to improve whole genome assemblies [30], this work, and work previously reported [3], [50] paves the way for their use in assembly-free bioinformatic approaches to provide a unique insight into the origin and structure of satellite repeats.

## CRediT authorship contribution statement

**Tihana Vondrak:** Investigation, Formal analysis, Software, Writing – Original Draft, Writing - review & editing. **Ludmila Oliveira:** Investigation, Writing - review & editing. **Petr Novák:** Formal analysis, Writing - review & editing. **Andrea Koblížková:** Resources. **Pavel Neumann:** Formal analysis, Writing - review & editing. **Jiří Macas:** Conceptualization, Supervision, Investigation, Writing – Original Draft, Writing - review & editing.

## Declaration of Competing Interest

The authors declare that they have no known competing financial interests or personal relationships that could have appeared to influence the work reported in this paper.
