# Supplementary material for: Complex sequence organization of heterochromatin in the holocentric plant Cuscuta europaea elucidated by the computational analysis of nanopore reads
Source: Comput Struct Biotechnol J. 2021 Apr 22;19:2179–89. doi: 10.1016/j.csbj.2021.04.011 (PMC8091179; doi:10.1016/j.csbj.2021.04.011)
Supplement: Supplementary data 1 [file mmc1.pdf]

Supplementary Fig. S1 A,B

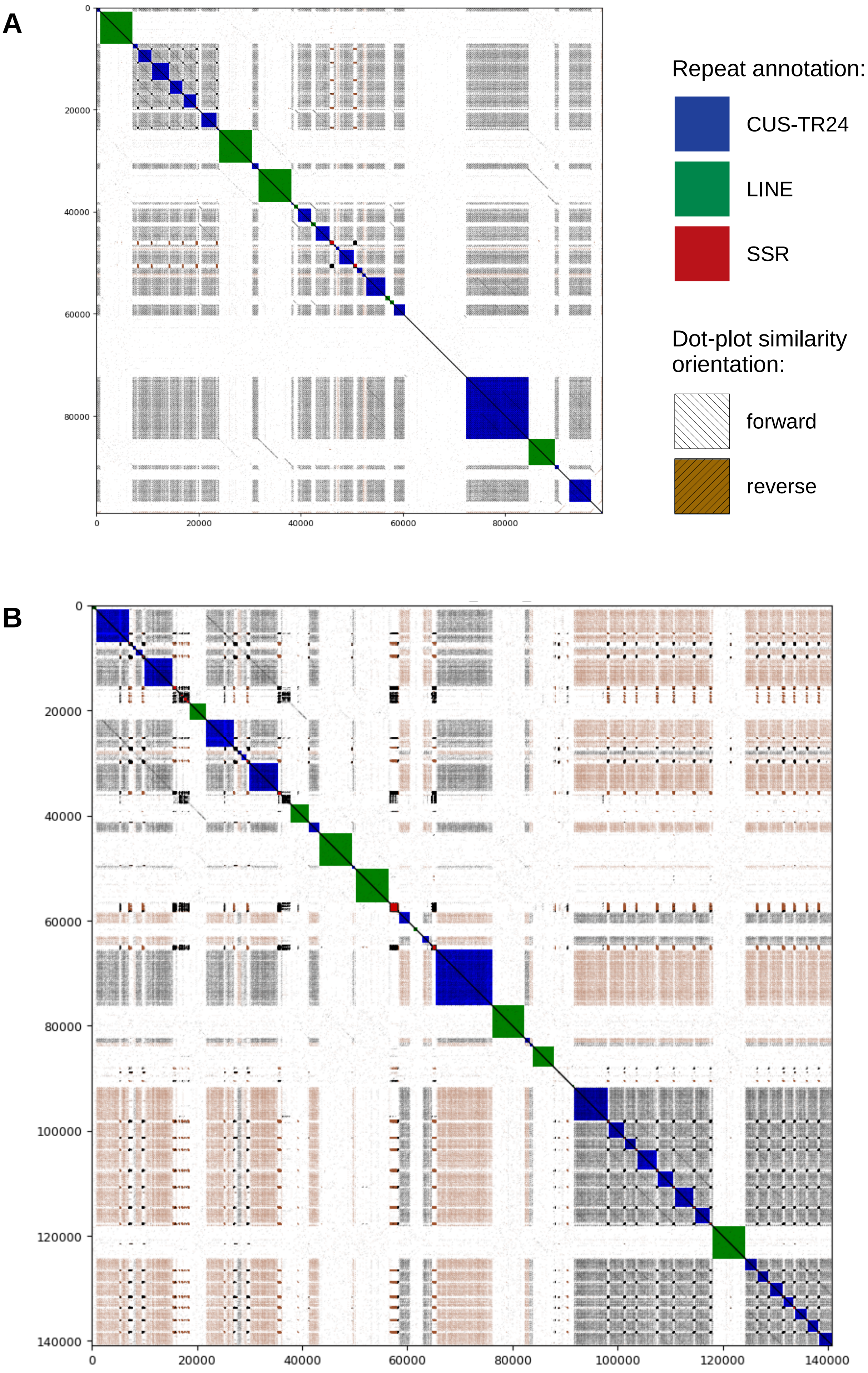

Supplementary Fig. S1 C,D

C

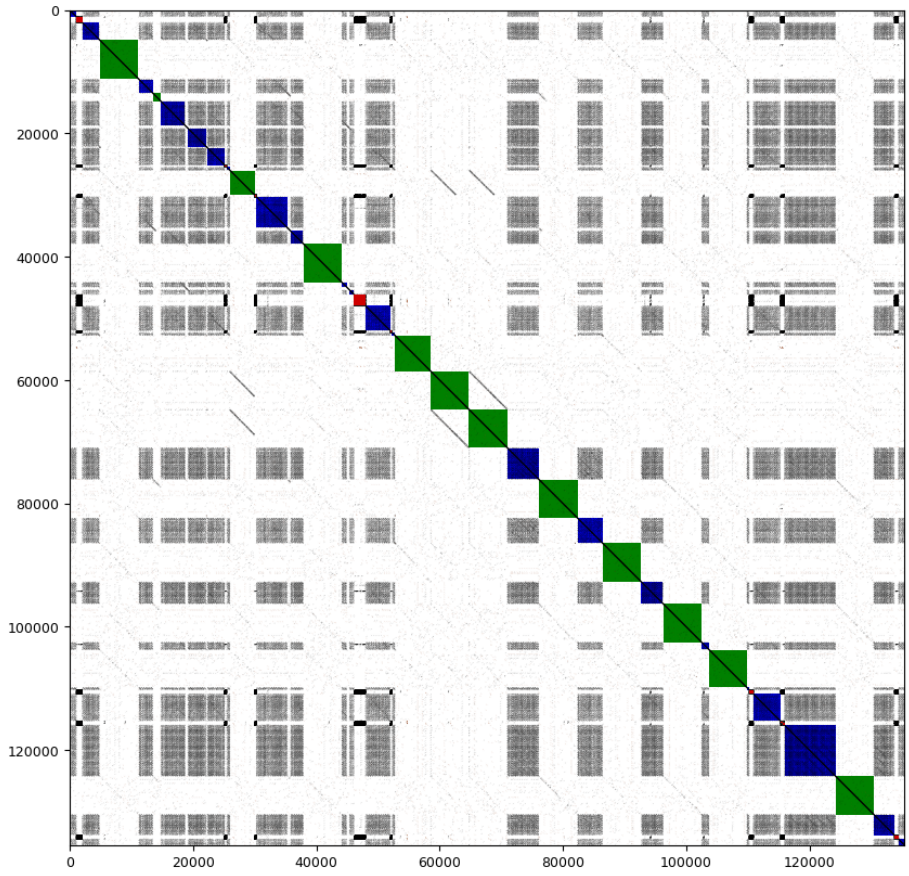

D

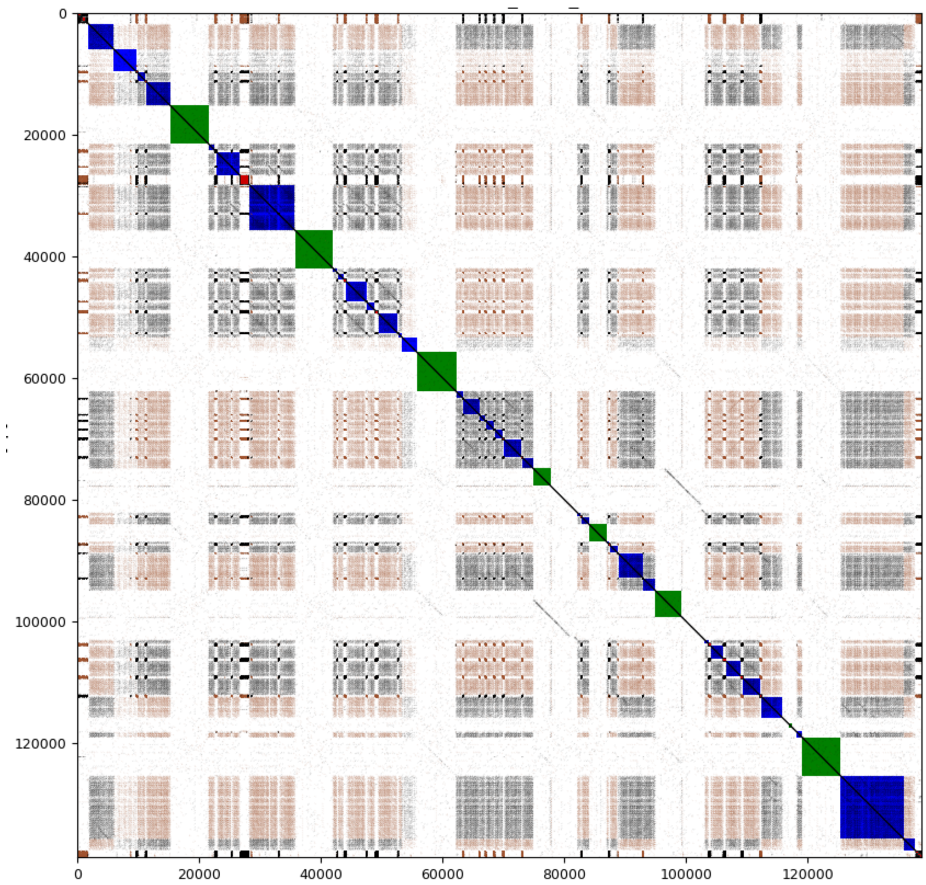

Supplementary Fig. S1. Examples of self-similarity dot-plots of individual nanopore reads (A-D) containing CUS-TR24 arrays. The plots were generated and annotated using FlexiDot (Seibt et al., 2018). The read used to generate dot-plot for Fig. 2 is shown on panel (A).

Supplementary Fig. S2

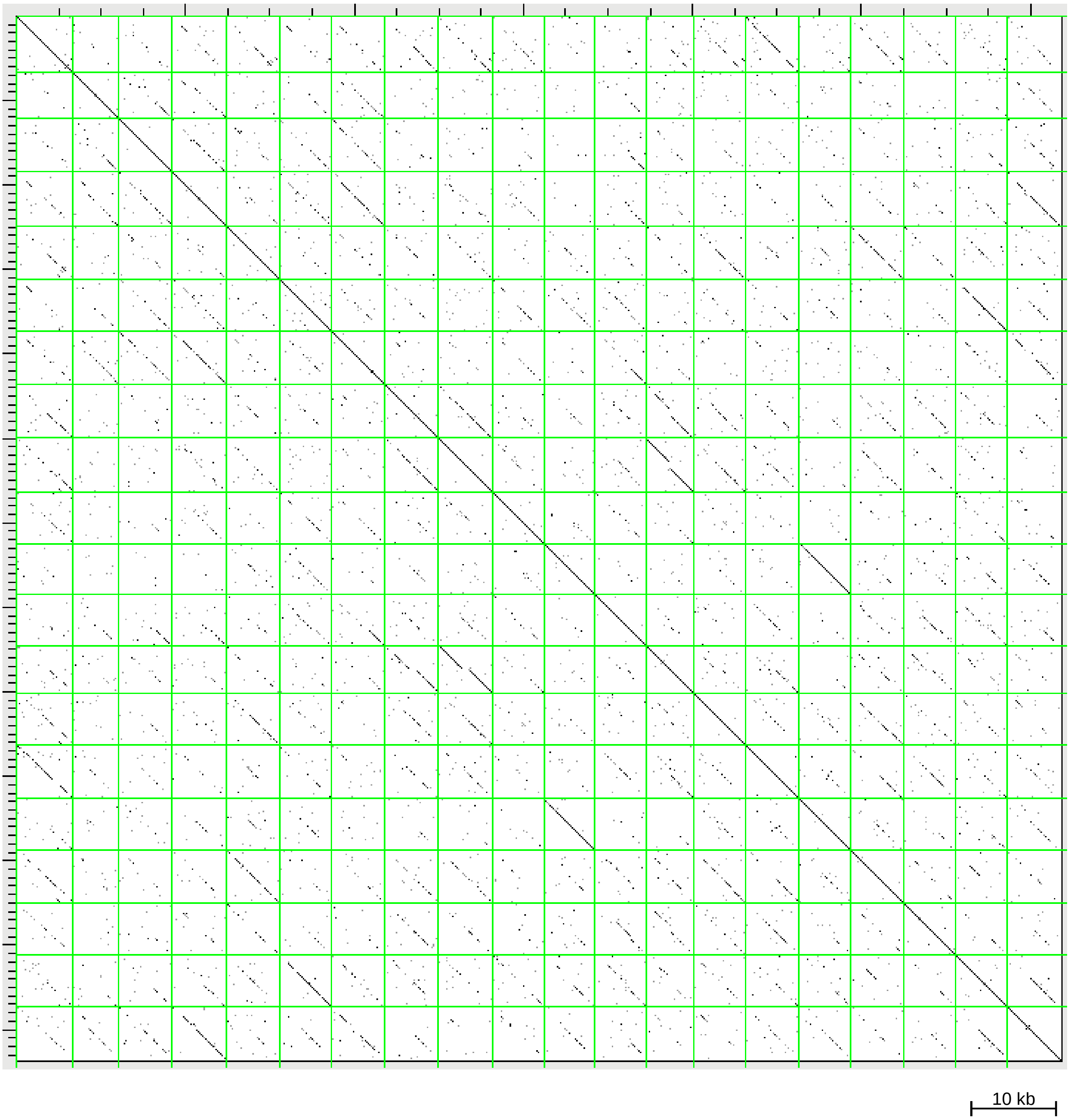

Supplementary Fig. S2. Sequence similarities of L1-CS LINE elements. The dot-plot shows all-to-all sequence comparison of twenty randomly sampled elements (the elements are separated by green lines). The similarities were scored within a sliding window of 100 bp and dots or lines were drawn when at least 80 matching bases were detected.

Supplementary Fig. S3

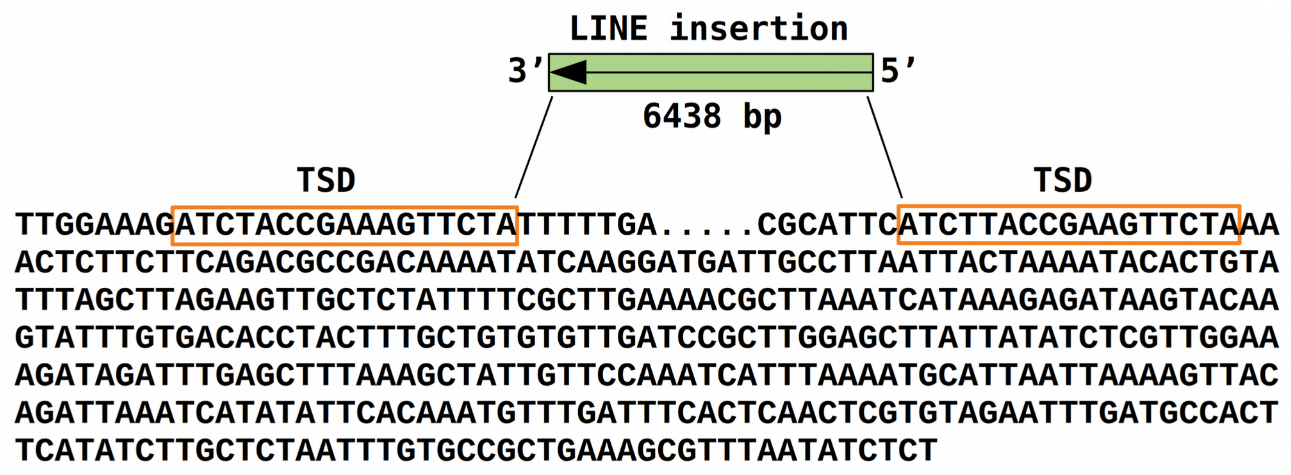

Supplementary Fig. S3. Example of the target site duplication (TSD) generated upon insertion of LINE element into CUS-TR24 monomer. The sequence was retrieved from a nanopore read, therefore the CUS-TR24 monomer sequence differs from the consensus provided in Fig. 8A. Only 5’ and 3’ terminal sequences are shown for the LINE element.
